# Berlin

**Published:** 1855

**Authors:** 


### BERLIN.

THE following is the Report of Meteorology and Epidemics in Berlin during the months of November and December, 1854:—

*November.* Frost occurred from the 10th day of the month to the end. The following extremes of temperature were observed:—

| | | | | | | | |
|---|---|---|---|---|---|---|---|
| At 7 a.m. | from | 46°·6F. | on the | 2nd, | to | 16°·25F. | on the 15th. |
| At 2 p.m. | „ | 50°·25F. | „ | 1st, | „ | 28°·7F. | „ 22nd. |
| At 9 p.m. | „ | 47°F. | „ | 1st, | „ | 24°F. | „ 14th. |

Snow fell on the 10th, 12th, 18th, 20th, 23rd, 28th, and 29th days of the month. Rain on the 30th. Fogs prevailed on the mornings of the 24th, 25th, and 28th. The wind was north on the 12th, 13th, and 19th; south on the 23rd and 28th; east on the 18th and 22nd; and west on the 10th, 11th, 14th, 15th, 16th, 20th, 21st, to 24th. 27th, and 30th. In the early part of the month, the west wind prevailed, and was especially stormy on the 4th.

Catarrhal fever, and catarrhal inflammation of the mucous membrane of the air passages and eyes, were very frequent, as were also rheumatic disorders. Acute articular rheumatism was especially frequent at the latter half of the month. Next in frequency was gastric fever, which was generally removed, under proper treatment, by the end of the second week. Ague was prevalent; some of the cases being relapses, and others fresh cases. The type was various, but more frequently tertian than quartan, and not unfrequently it was very irregular. Pneumonia, conjoined with pleurisy, was the most common form of inflammatory disease; it was generally sthenic, and admitted, for the most part, of successful treatment. Several cases of scarlet fever and measles had appeared; and it appeared likely that there would be an epidemic of the latter disease. Erysipelas, principally of the face, attacked several persons. In chronic diseases, the influence of the weather was very remarkable, especially in phthisis, the patients labouring under which disease had their sufferings much increased, and were frequently troubled with spitting of blood. Patients affected with dropsies, chronic catarrh, and emphysema of the lungs, were much distressed. Apoplexy also became more frequent than in the preceding months.

Among domestic animals, horses suffered chiefly from catarrhal affections in the early part of the month, but in the latter, more from rheumatic affections. Among bovine cattle, acute rheumatism frequently occurred, with rheumatic gastric fever, and some cases of pleuropneumonia. Among the diseases of sheep, goats, and swine, nothing remarkable was observed. Dogs suffered from various rheumatic and catarrhal diseases; there were no cases of canine madness.

*December.*—The unfavourable weather which prevailed towards the end of November continued through the whole of December. Frost was very slight, and occurred only on the 6th, 7th, 8th, 11th, 13th, 18th, 19th, 20th, 21st, 27th, 28th, and 29th. The 6th, 11th, and 29th were the only tolerably fair and bright days.

The following were the variations in the temperature observed during the month:—

| | | | | | | |
|---|---|---|---|---|---|---|
| At 7 a.m. | from 42°F. | on the | 4th, | to 36°·5F. | on the | 21st. |
| At 2 p.m. | „ 47°·75F. | „ | 15th, | to 28°·8F. | „ | 12th. |
| At 9 p.m. | „ 39°F. | „ | 4th, | to 26°F. | „ | 12th. |

The temperature was extraordinarily mild through the whole month, but there was also a considerable degree of humidity. Rain, with varied proportions of hail and snow, fell on the 1st, 4th, 5th, 7th, 8th, 9th, 10th, 14th, 15th, 16th, 17th, 18th, and from the

22nd to the 28th, and again on the 31st. There were also slight falls of snow on the 2nd, 3rd, 9th, 13th, 19th, 20th and 21st. Fog prevailed on the morning of the 8th and 15th, and during the whole of the 12th. With the exception of a brief veering to the south on the 9th, 13th and 18th, and to the north on the 28th, the wind was west during the whole month; it was often violent; and from the 23rd to the end of the month was very stormy. There were short calms on the 7th, 11th, 12th, 21st and 29th.

The weather continued, as in November, to exercise an unfavourable influence on the general health. Catarrhal disorders were unusually frequent; and there were few persons who passed through the month without more or less interruption to their general health. The prevailing sickness was catarrhal fever, with coryza, cough, and hoarseness with slight quinsy; rheumatic disorders were next in frequency. Besides the more severe forms of acute articular rheumatism, slight forms of muscular rheumatism and rheumatic neuralgia were observed, especially in the teeth. Gouty and chronic rheumatic affections, as also chronic nervous diseases, were much aggravated under the influence of the inclement weather and the cloudy, murky sky. Convalescence from acute disorders was also greatly retarded.

The severity of the diseases was not in proportion to the number of cases. With the exception of some single cases of membranous croup, occurring in predisposed children, and some cases of acute bronchitis and pneumonia in grown persons, there were no dangerous diseases. Gastric and gastro-nervous fever was certainly less frequent than is usual at this time of the year. Cases of intermittent fever were as frequent as usual, evidently for the most part made up of relapses. Measles had not extended; and some single bad cases of scarlet fever were noticed.

In spite of the frequent changes in the weather, domestic animals suffered only moderately, and chiefly from rheumatic affections. Horses suffered pretty frequently from acute rheumatism, rheumatic fever, and rheumatic inflammation in various parts; as well as of catarrh, catarrhal fever, quinsy, pleuropneumonia, influenza, inflammation of the stomach and intestines, colic, grease, inflammation of the lymphatics, tetanus, &c. The bovine cattle suffered chiefly from rheumatism, purulent inflammation, gastro-rheumatic fever, and in two places also from pleuropneumonia. Little was remarkable with regard to the diseases of sheep and swine. Dogs suffered much from rheumatic and catarrhal affections of the respiratory organs. One case of rabies occurred at Spandow, near Berlin.

*Medicinische Zeitung.*

